# Metal cations promote α-dicarbonyl formation in glucose-containing peritoneal dialysis fluids

**DOI:** 10.1007/s10719-020-09964-6

**Published:** 2020-12-07

**Authors:** Sabrina Gensberger-Reigl, Andrea Auditore, Jochen Huppert, Monika Pischetsrieder

**Affiliations:** 1 Food Chemistry, Department of Chemistry and Pharmacy, Friedrich-Alexander-Universität Erlangen-Nürnberg (FAU), Nikolaus-Fiebiger-Straße10, 91058 Erlangen, Germany; 2 Fresenius Medical Care Deutschland GmbH, Frankfurter Straße 6-8, 66606 St. Wendel, Germany

**Keywords:** Glucose degradation products (GDPs), α-Dicarbonyls, Peritoneal dialysis fluid, Metal cations

## Abstract

Heat sterilization of peritoneal dialysis fluids (PDFs) leads to the formation of glucose degradation products (GDPs), which impair long-term peritoneal dialysis. The current study investigated the effects of metal ions, which occur as trace impurities in the fluids, on the formation of six major α-dicarbonyl GDPs, namely glucosone, glyoxal, methylglyoxal, 3-deoxyglucosone, 3-deoxygalactosone, and 3,4-dideoxyglucosone-3-ene. The chelation of metal ions by 2-[bis[2-[bis(carboxymethyl)amino]ethyl]amino]acetic acid (DTPA) during sterilization significantly decreased the total GDP content (585 μM vs. 672 μM), mainly due to the decrease of the glucose-oxidation products glucosone (14 μM vs. 61 μM) and glyoxal (3 μM vs. 11 μM), but also of methylglyoxal (14 μM vs. 31 μM). The glucose-dehydration products 3-deoxyglucosone, 3-deoxygalactosone, and 3,4-dideoxyglucosone-3-ene were not significantly affected by chelation of metal ions. Additionally, PDFs were spiked with eleven different metal ions, which were detected as traces in commercial PDFs, to investigate their influence on GDP formation during heat sterilization. Iron(II), manganese(II), and chromium(III) had the highest impact increasing the formation of glucosone (1.2–1.5 fold increase) and glyoxal (1.3–1.5 fold increase). Nickel(II) and vanadium(III) further promoted the formation of glyoxal (1.3 fold increase). The increase of the pH value of the PDFs from pH 5.5 to a physiological pH of 7.5 resulted in a decreased formation of total GDPs (672 μM vs 637 μM). These results indicate that the adjustment of metal ions and the pH value may be a strategy to further decrease the content of GDPs in PDFs.

**Supplementary Information:**

The online version contains supplementary material available at 10.1007/s10719-020-09964-6.

## Introduction

Reactive glucose degradation products (GDPs) are of high relevance for the production of glucose-containing drugs. Usually, products such as peritoneal dialysis fluids (PDFs) or infusion solutions are heat-sterilized to ensure the microbiological safety. As a consequence, however, a variety of reactive GDPs are formed [1]. Most GDPs contain an α-dicarbonyl moiety that reacts readily with nucleophilic side chains of amino acids such as lysine, arginine, or cysteine to form advanced glycation products (AGEs) [2–4]. AGEs are responsible for a critical loss of protein function, such as the impairment of enzymatic activity or receptor binding [5–8]. Especially in the stable low-turnover protein collagen, cross-linking AGEs can increase the stiffness of the collagen network and, for example, cause brittleness [9, 10]. Thus, changes in membrane morphology, membrane thickening, and a declining ultrafiltration capacity of the peritoneal membrane were associated with a high GDP load in PDFs and AGE formation [11–14]. These complications can eventually cause the discontinuation of this renal replacement therapy. Moreover, α-dicarbonyls are cytotoxic to human mesothelial cells or fibroblasts [8, 15]. These effects were linked to the denudation of mesothelial cells in the peritoneum, which was observed in vivo during the long-term application of PDFs [12].

**Fig. 1:**
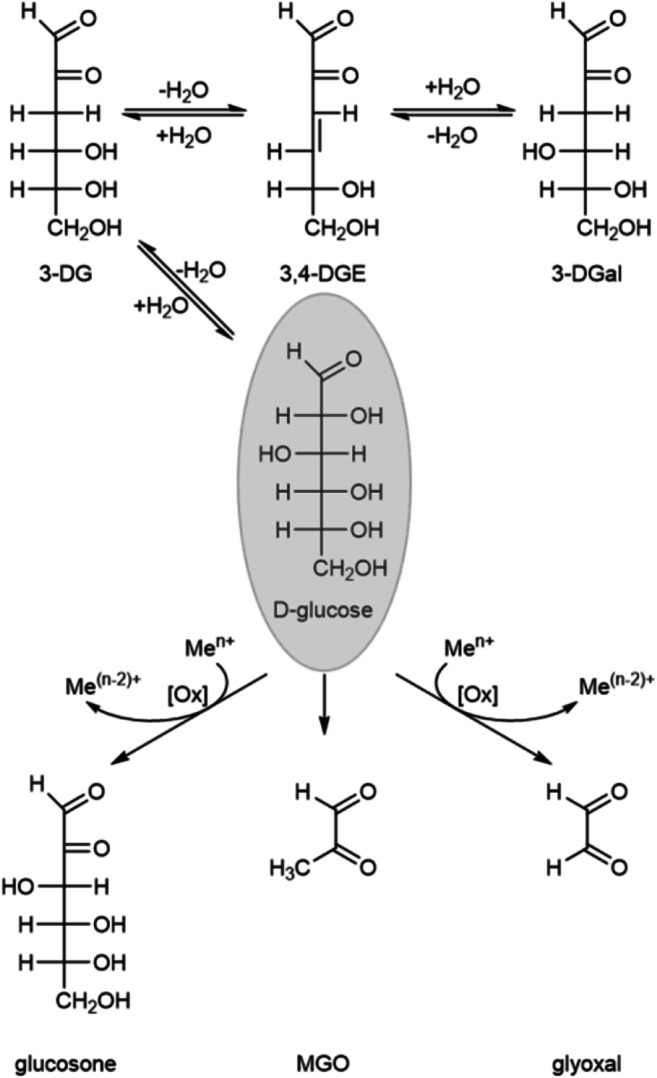
GDPs with α-dicarbonyl structure formed during heat sterilization of PDFs; Ox, oxidation

In glucose-containing drugs, the α-dicarbonyls glucosone, 3-deoxyglucosone (3-DG), 3-deoxygalactosone (3-DGal), glyoxal, 3,4-dideoxyglucosone-3-ene (3,4-DGE), and methylglyoxal (MGO) were identified (Fig. 1) [1, 16]. In total, up to 450 μM GDPs with α-dicarbonyl structure were detected in commercial PDFs [17]. A better understanding of the reaction mechanisms underlying GDP formation in this matrix is important to improve the biocompatibility of PDFs. The formation of α-dicarbonyls depends on the product composition. Different factors like the pH value, the concentration of the raw materials, storage periods, or oxygen permeability of the packaging material play a role in glucose degradation [18–21]. Additionally, it can be assumed that impurities in the raw materials may also influence the GDP content of the product. For instance, metal cations could catalyze the oxidative glucose degradation [22–24].

The goal of the present study was therefore to investigate the influence of metal ions on the formation of six major glycating agents with α-dicarbonyl moieties, namely 3-DG, 3-DGal, 3,4-DGE, glucosone, glyoxal, and MGO, in PDF matrices. For this purpose, PDFs were spiked one at a time with eleven different metal cations representing the most prominent impurities in commercial products. After heat sterilization, comprehensive α-dicarbonyl profiling by ultra-high performance liquid chromatography coupled with diode array detection was performed to quantify 3-DG, 3-DGal, 3,4-DGE, glucosone, glyoxal, and MGO.

## Material and methods

### Reagents

Unless noted otherwise, all chemicals were obtained from Sigma-Aldrich. LCMS-grade methanol (Fisher Scientific, Schwerdte, Germany), formic acid (VWR, Darmstadt, Germany), ammonium formate and purified water from a Synergi-185 labwater system (Millipore, Schwalbach, Germany) were used for all experiments. d-Glucose monohydrate, sodium-l-lactate, sodium chloride, magnesium chloride hexahydrate, and calcium chloride dihydrate complied with the requirements of the European Pharmacopoeia and the ICH guideline Q3D (R1) on elemental impurities [25, 26]. Glucosone [27], 3-DGal [28], and 3,4-DGE [17] were synthesized as reported previously. 3-DG was purchased from Chemos (Altdorf, Germany), 4-(2-hydroxyethyl)-1-piperazineethanesulfonic acid (HEPES) from Carl Roth (Karlsruhe, Germany), and hydrochloric acid (pro analysi) as well as sodium hydroxide (purity 99%) from Grüssing (Filsum, Germany). Eleven different inorganic salts were tested (all obtained from Sigma-Aldrich): lithium(I) chloride (purity ≥99.0%), aluminum(III) sulfate (99.99%), vanadium(III) chloride (97%), chromium(III) chloride hexahydrate (≥ 98%), manganese(II) chloride tetrahydrate (≥ 99.0%), iron(II) chloride (98%), iron(III) chloride (≥ 99.9%), nickel(II) chloride hexahydrate (99.99%), copper(II) chloride dihydrate (99.99%), zinc(II) chloride (99.99%), and molybdenum(IV) oxide (99%).

### Quantification of metal impurities in commercial PDFs

The average concentration of metal impurities in commercial PDFs was determined by inductively coupled-plasma mass spectrometry (ICP–MS) [29]. Molybdenum and aluminum were not included in the screening of commercial PDFs, but analyzed in raw materials. The molybdenum and aluminum contents in PDFs were then calculated based on the amount of the raw materials in the PDFs.

### Sample preparation

Unsterilized PDFs were prepared containing 7.8 g/L sodium l-lactate, 101.7 mg/L magnesium chloride hexahydrate, 257.3 mg/L calcium chloride dihydrate, 5.8 g/L sodium chloride and 4.25% glucose. Some PDF samples were supplemented with 2-[bis[2-[bis(carboxymethyl)amino]ethyl]amino]acetic acid (DTPA, final concentration 3.88 mM). DTPA chelates inorganic cations, which are present as trace impurities. The pH of the PDF samples was adjusted to 5.5 or 7.5 with hydrochloric acid or sodium hydroxide, respectively. Additionally, solutions containing 101.7 mg/L magnesium chloride hexahydrate, 257.3 mg/L calcium chloride dihydrate, 5.8 g/L sodium chloride and 4.25% glucose each were prepared with and without 3.88 mM DTPA to model the acidic compartment of a double-chamber bag PDF. The pH was adjusted to 3.1 with hydrochloric acid.

Aqueous stock solutions of the different inorganic salts were prepared in volumetric flasks: lithium(I) chloride 0.76 mg/mL, aluminum(III) sulfate 0.28 mg/mL, vanadium(III) chloride 0.14 mg/mL, chromium(III) chloride hexahydrate 0.25 mg/mL, manganese(II) chloride tetrahydrate 0.14 mg/mL, iron(II) chloride 0.39 mg/mL, iron(III) chloride 0.49 mg/mL, nickel(II) chloride hexahydrate 0.83 mg/mL, copper(II) chloride dihydrate 0.46 mg/mL, zinc(II) chloride 0.13 mg/mL, and molybdenum(IV) oxide 0.90 mg/mL. The stock solutions were diluted with ultra-purified water. The PDFs were spiked with one of the eleven different salt solutions each to a metal concentration of approximately ten times the average in commercial products. Table 1 lists the final cation concentrations. The pH of these solutions was adjusted to 5.5 or 7.5 after spiking.

**Table 1:** Metal concentrations in commercial PDFs and the concentrations of metal ions used for spiking experiments. The metal concentration levels of 26 different commercial PDFs were analyzed by ICP–MS.

	metal concentrations in commercial PDFs [ppb]		final cation concentration in spiked PDFs [ppb]
Li	0.04–0.07	Li^+^	0.6
Al	0.01–0.02^a^	Al^3+^	0.2
V	0.02–0.13	V^3+^	0.2
Cr	2.14–2.38	Cr^3+^	24.2
Mn	1.61–1.76	Mn^2+^	19.9
Fe	7.28–8.21	Fe^2+^	85.0
		Fe^3+^	83.7
Ni	0.86–1.01	Ni^2+^	10.3
Cu	0.07–0.09	Cu^2+^	0.9
Zn	0.14–0.47	Zn^2+^	3.2
Mo	0.003–0.004^a^	Mo^4+^	0.03

^a^calculated values based on concentrations in the raw materials of the PDF

Unspiked PDFs served as negative controls. All PDFs were heat-sterilized (121 °C for 45 min) in a laboratory autoclave (SHP Steriltechnik, Detzel Schloss/Satuelle, Germany), which is equivalent to the industrial sterilization of PDFs. The α-dicarbonyl GDPs were quantified after cooling down to room temperature. All experiments were performed in triplicates.

### Stability of GDPs prior to derivatization

The stability of the GDPs in the different PDF matrices was assessed to exclude bias by degradation processes after the sterilization step, but prior to derivatization. For that purpose, unsterilized glucose-free PDF solutions were prepared at pH 5.5 and 7.5, with and without DTPA, respectively. The four samples were spiked with glucosone, glyoxal, methylglyoxal, 3-deoxyglucosone, 3-deoxygalactosone, and 3,4-dideoxyglucosone-3-ene in aqueous solution and derivatized immediately after preparation or after resting for 15 and 30 min at room temperature. The experiments were performed in triplicates and showed that the six GDPs were stable at pH 5.5 and pH 7.5 in the presence and absence of DTPA (Supplementary Fig. S1).

### Quantitative GDP profiling by ultra-high performance liquid chromatography–diode array detection

GDP profiling was performed as previously reported [17]. Briefly, α-dicarbonyls were derivatized with *o*-phenylenediamine yielding their respective quinoxaline derivatives. The pH of the double-chamber bag model was adjusted to 5.5 prior to derivatization. A Thermo Fisher UltiMate 3000RS liquid chromatography system consisting of a pump with degasser, autosampler, column compartment, and diode array detector equipped with an ACQUITY UPLC® BEH phenyl column (1.7 μm particle size; 2.1 × 100 mm, Waters, Eschborn, Germany) was used for the chromatographic separation of the quinoxalines. The system was controlled by Chromeleon 6.80 (Thermo Fisher Scientific, Dreieich, Germany). The quinoxaline derivatives were analyzed between 120 and 650 min after adding the derivatizing reagent.

## Results and discussion

The main purpose of the present study was to investigate the impact of inorganic cations, which are observed as impurities in PDFs, on the heat-induced formation of the six major α-dicarbonyl GDPs in PDFs.

### Analysis of GDPs in unheated PDFs

It has been reported that glucose contains basal levels of glucosone and 3-DG [17, 27, 28]. To quantify any GDPs introduced by the matrix, unheated aliquots of all PDFs were screened for α-dicarbonyls. Neither 3-DGal, glyoxal, 3,4-DGE, nor MGO were detectable, but low amounts of glucosone (13.1 μM ± 0.7 μM) and 3-DG (16.0 μM ± 0.1 μM) were present in all unheated PDFs. The GDP content in the unheated samples was not influenced by the addition of inorganic cations or DTPA (glucosone: 12.2 ± 0.1 μM without DTPA and 12.0 ± 0.1 μM with DTPA, 3-DG 15.7 ± 0.04 μM without DTPA and 15.8 ± 0.02 μM with DTPA; Supplementary Fig. S2).

The complete and reproducible derivatization of all GDPs is a prerequisite for reliable quantification, but de novo formation of GDPs like glucosone and glyoxal has been reported after derivatization with *o*-phenylenediamine in glucose-rich matrices [23, 27, 30], depending on the sugar content and composition of the matrix [28, 31]. In the present study, the possible de novo formation of GDPs during derivatization was examined by quantifying the GDPs in unheated PDFs with or without metal ions or DTPA and during progressing derivatization, respectively. The content of all GDPs was constant between 120 and 650 min after adding *o*-phenylenediamine. Thus, de novo formation of glucosone and glyoxal as a derivatization artifact could be excluded (Supplementary Fig. S2). The results confirmed that the applied derivatization procedure is suitable for reliable α-dicarbonyl profiling in PDF matrices even in the presence of spiked metal ions.

### Influence of metal ion traces in PDFs on the GDP formation during heat sterilization at pH 5.5

To investigate the influence of metal ion traces from raw materials on the GDP formation in PDFs during heating, the samples were sterilized in the presence and absence of DTPA. DTPA is a strong chelating agent for mono- and bivalent cations reducing their catalytic activity.

**Fig. 2:**
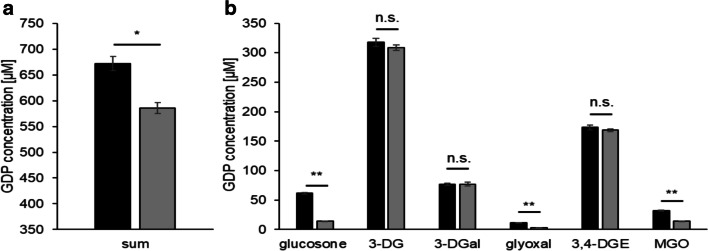
Concentrations of (a) total and (b) individual GDPs in heat-sterilized PDFs containing 4.25% glucose and conventional electrolytes at pH 5.5 without (black bars) and with DTPA (gray bars). Mean values ± standard deviations of triplicates are displayed. A two-tailed, paired t-test was calculated to show differences between the samples (n.s. not significant, * *p* < 0.05, ** < p 0.01)

When metal cations were chelated by DTPA during the sterilization process at pH 5.5, the total GDP load decreased significantly from 672 μM to 585 μM (Fig. 2a). The overall decrease was mainly due to glucosone (61 μM vs. 14 μM), glyoxal (11 μM vs. 3 μM), and MGO (31 μM vs. 14 μM). The concentrations of 3-DG, 3-DGal, and 3,4-DGE, however, were not significantly influenced by DTPA (Fig. 2b). The glucosone content of the solution with DTPA was almost equal to the amount in unheated PDFs. Our results indicate that metal traces, which are transferred to the product as impurities in the raw materials or from production equipment made of stainless steel, enhance glucose degradation during the heat sterilization of conventional PDFs. The formation of glucosone seems to be completely dependent on the presence of metal ions.

It is well established that glucosone and glyoxal are formed by oxidative glucose degradation [32–34], whereas 3-DG, 3-DGal, and 3,4-DGE mainly derive from dehydration reactions [35, 36]. Formation of MGO is mostly described by a retro-aldol cleavage of glucose via the 2,3-endole intermediate [32]. The data obtained here indicate that MGO can be additionally formed at pH 5.5 via a metal-catalyzed oxidative pathway. Chelating agents, which mask metal cations, may reduce the GDP load in PDFs.

### Influence of specific metal ions on the GDP formation at pH 5.5

To determine the most relevant metal ions for the GDP formation, the test solutions were spiked with different inorganic salts prior to heat sterilization. Eleven metal cations were chosen for analysis after screening commercial PDFs by ICP–MS for common metal impurities [29]. ICP–MS, however, does not provide information on the oxidation state of the metals. Thus, we used the most common and stable forms, which were available as chloride or sulfate salts at very high purities. Iron was applied at two different oxidation states, namely iron(II) and iron(III) and molybdenum(IV) was used as dioxide. To delineate the specific effects, each metal ion was added to the test solution in a concentration that was nearly ten times higher than its basal concentration in the conventional PDFs (Table 1).

**Fig. 3:**
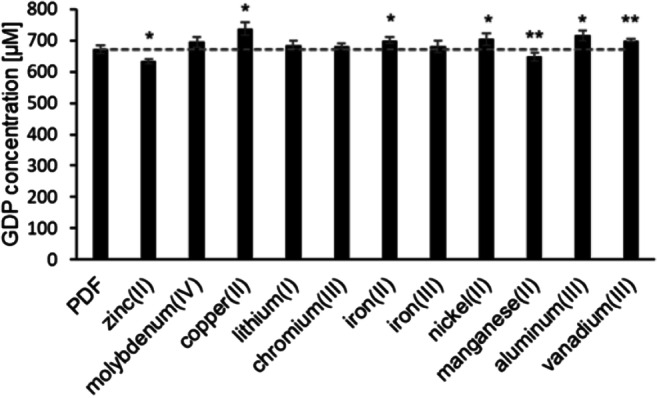
Concentrations of total GDPs in an unspiked PDF (pH 5.5, 4.25% glucose; control) and in the same fluid spiked with different metal cations. Each metal ion was added in a tenfold concentration compared to the respective basal content in PDFs. Mean values ± standard deviations of triplicates are displayed. A two-tailed, paired t-test was calculated to show differences between the samples and the conventional PDF (control) (* *p* < 0.05, ** *p* < 0.01). The dashed line shows the GDP level of the control fluid

The total α-dicarbonyl content in PDFs (pH 5.5) spiked with inorganic salts ranged from 634 μM to 737 μM. When copper(II), iron(II), nickel(II), aluminum(III), or vanadium(III) were added, the total GDP contents were significantly higher compared to the unspiked PDF. In contrast, molybdenum(IV), lithium(I), chromium(III), or iron(III) did not change the total α-dicarbonyl load. PDFs spiked with zinc(II) or manganese(II) contained less α-dicarbonyls (Fig. 3). Since the total GDP concentration may mask individual variations, decreases or increases of the contents in the spiked solutions were monitored separately for each single α-dicarbonyl compound.

**Fig. 4:**
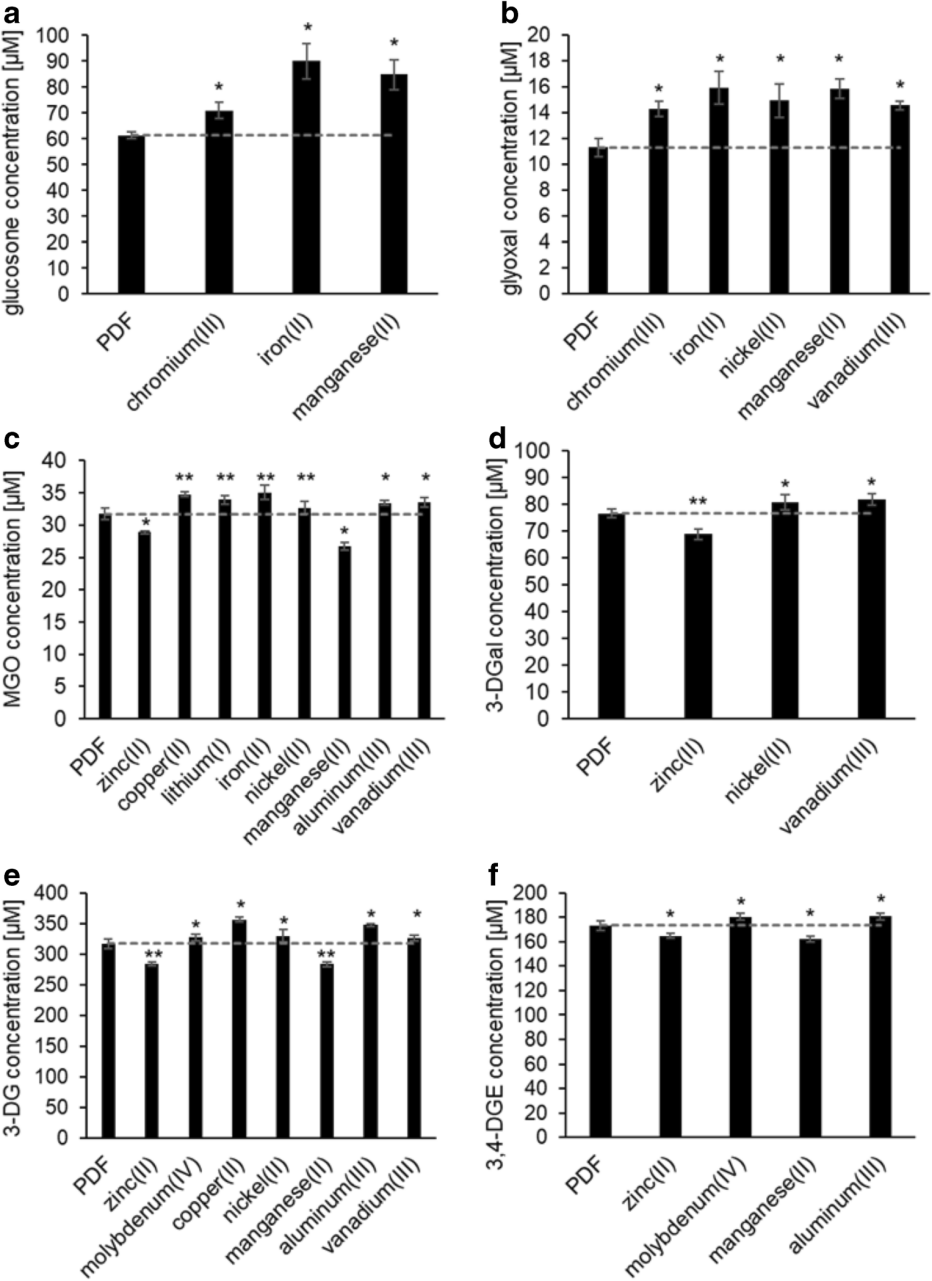
Concentrations of individual GDPs in an unspiked PDF (pH 5.5, 4.25% glucose; control) and the same fluid spiked with different metal cations. Mean values ± standard deviations of triplicates are displayed. A two-tailed, paired t-test was calculated to show differences between the samples and the unspiked PDF (control) (* p < 0.05, ** p < 0.01). The dashed line shows the GDP level of the control fluid. Results with non-significant differences are displayed in the electronic supplementary material (Fig. S3)

The glucosone content of the PDFs supplemented with inorganic cations ranged from 60 μM to 90 μM. Additional chromium(III), iron(II), and manganese(II) enhanced the glucosone formation significantly compared to the control (Fig. 4a). No changes were detected for lithium(I) and vanadium(III). For all other inorganic cations, a slight, but statistically not significant increase could be observed (Supplementary Fig. S3a). Glucosone is formed via oxidation of glucose. It has been shown that pro-oxidative metal cations can catalyze glucose autoxidation [22, 24, 37] and, thus, promote the formation of glucosone. Our data indicate that chromium(III), iron(II), and manganese(II) are the most relevant inorganic impurities promoting glucosone formation in conventional PDFs. The observation that iron(II), but not iron(III), enhanced glucosone formation indicates a Fenton reaction. Thus, it can be assumed that the actual oxidizing agents are reactive oxygen species such as hydrogen peroxide or superoxide [38].

Interestingly, the addition of metal ions in a tenfold higher concentration compared to the basal levels in PDFs raised the glucosone concentration only by a relatively low factor of maximal 1.5 (iron(II), 61 vs. 90 μM). In contrast, the removal of basal metal ions by DTPA resulted in a 4.4-fold lower glucosone concentration (61 vs. 14 μM), This observation indicates that the concentration of metal ions is not necessarily rate-limiting for the reaction. It has been suggested before, for example, that the content of dissolved oxygen limits the formation of glucosone from glucose in PDFs [27]. Another factor can be the partial stabilization of glucose by complexation with higher concentrations of metals or additive/synergistic effects of different metal ions.

Glyoxal was present in concentrations between 11 μM and 16 μM. Even if the absolute differences were rather low, significantly more glyoxal was formed when chromium(III), iron(II), nickel(II), manganese(II), or vanadium(III) were added (Fig. 4b). A slight, but not significant increase could be observed for all other metal ions (Supplementary Fig. S3b). For glyoxal, different formation pathways have been described. Firstly, direct formation of glyoxal via retro-aldol cleavage of glucose was suggested [32]. Secondly, it was proposed that glyoxal is formed via further degradation of glucosone by C2–C3 cleavage [33, 34]. Thirdly, glyoxal can be also formed by dehydration of glucose and subsequent retro-aldol cleavage between C2–C3 [39]. Our results show that metal cations contribute to the formation of glyoxal in PDFs indicating an oxidative reaction mechanism in PDFs. Additional formation pathways, however, cannot be excluded.

The MGO content of all PDFs varied from 27 μM to 35 μM. Except for samples containing molybdenum(IV), chromium(III), and iron(III) (Supplementary Fig. S3c), statistically significant differences were observed between the conventional PDF and the spiked samples (Fig. 4c). The presence of copper(II), lithium(I), iron(II), nickel(II), aluminum(III), or vanadium(III) enhanced MGO formation indicating that these cations contribute to the oxidative degradation of glucose to this GDP. Interestingly, significantly less MGO was formed when zinc(II) and manganese(II) were present (Fig. 4c).

The concentration levels of 3-DGal ranged between 69 μM and 82 μM. Most inorganic cations did not influence the 3-DGal formation (Supplementary Fig. S3d) except for vanadium(III), nickel(II), and zinc(II). Vanadium(III) and nickel(II) promoted the formation of 3-DGal, whereas zinc(II) ions led to a slight decrease of the 3-DGal content (Fig. 4d). 3-DG was the main α-dicarbonyl in all PDFs. Its content ranged from 284 μM to 357 μM. Additional zinc(II) and manganese(II) caused a significant decrease of the 3-DG content, while molybdenum(IV), copper(II), nickel(II), aluminum(III), or vanadium(III) effected a significant increase (Fig. 4e). All other metal ions did not influence the 3-DG content (Supplementary Fig. S3e). The amount of 3,4-DGE ranged from 162 μM to 182 μM. Copper(II), lithium(I), chromium(III), iron(II), iron(III), nickel(II), or vanadium(III) did not influence 3,4-DGE significantly (Supplementary Fig. S3f). Aluminum(III) and molybdenum(IV), however, caused an increase, manganese(II) and zinc(II) a decrease of the 3,4-DGE contents compared to the control (Fig. 4f).

While metal cations can influence the formation of 3-DG, 3-DGal, or 3,4-DGE, the absolute differences in concentration were rather low. Thus, metal cations are of lower relevance for the GDP formation by non-oxidative pathways. In contrast, they seem of high relevance for GDPs which are formed by oxidative pathways. In general, metal ions can influence GDP formation by different mechanisms depending on the metal and the GDP. Besides direct oxidation or Fenton reaction, metal ions could also be involved as catalyzer or chelating agents. Further studies are required to elucidate the mechanisms of the individual effects. Additionally, the activity of alternative metal species that may occur in water should be assessed, mainly chromate or vanadate.

### Influence of pH on the GDP formation

**Fig. 5:**
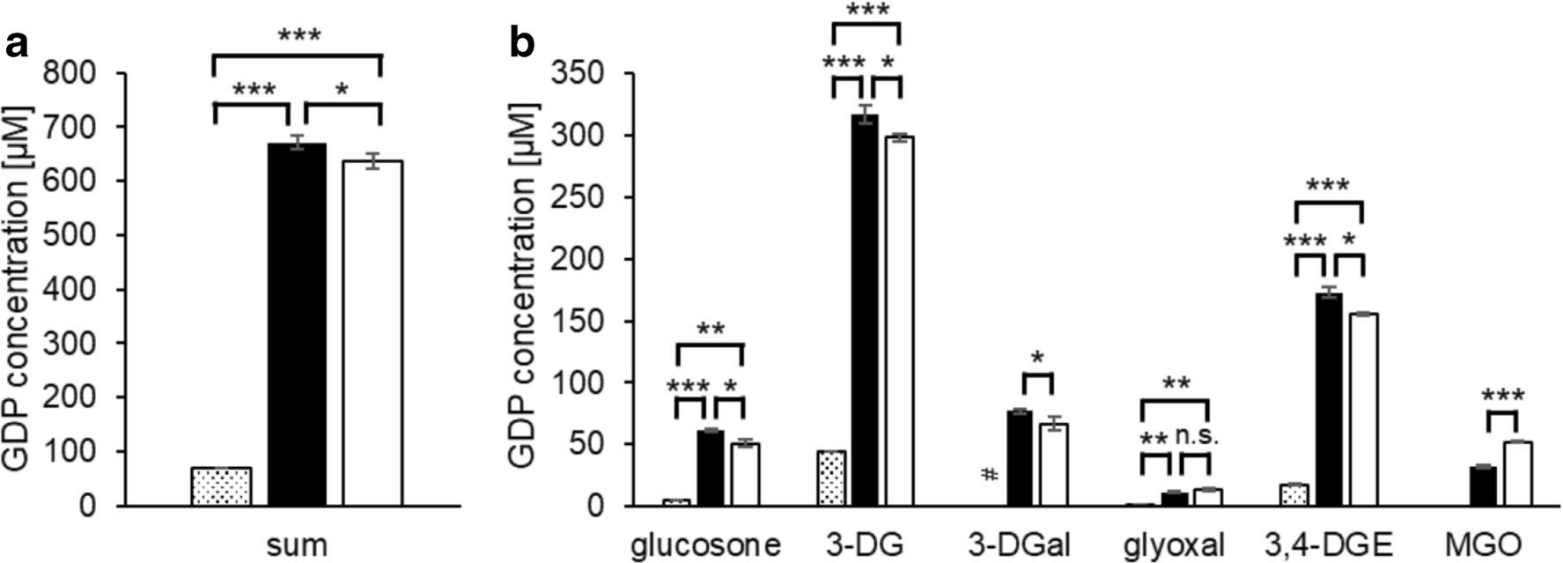
Concentrations of (a) total and (b) individual GDPs in heat-sterilized PDFs containing 4.25% glucose and conventional electrolytes at pH 3.1 (dotted bars), pH 5.5 (black bars), and pH 7.5 (white bars). Mean values ± standard deviations of triplicates are displayed. A two-tailed, paired t-test was calculated to show differences between the samples at pH 3.1, pH 5.5, and pH 7.5 (n.s. not significant, * p < 0.05, *** *p* < 0.001). ^#^ At pH 3.1, 3-DGal could not be quantified because of coelution of 5-hydroxmethylfurfural

Conventional lactate-buffered single-chamber PDFs have a slightly acidic pH of 5.5. Because the acidic pH reduces the biocompatibility of these products, PDFs with a physiological pH value and a low GDP load are preferred [40, 41]. We investigated the impact of pH on the GDP formation in PDF matrix, analyzing pH 5.5 and pH 7.5 as well as pH 3.1 as control. After heat treatment, the PDF at pH 5.5 contained significantly more GDPs (672 μM) in total than the PDF at pH 7.5 (637 μM) (Fig. 5a). In detail, the levels of glucosone, 3-DG, 3-DGal, and 3,4-DGE were higher at pH 5.5 than pH 7.5 (Fig. 5b). Zimmeck et al. analyzed the formation of 3-DG in relation to the pH value and reported a gradual increase of 3-DG with an increasing pH from 3.5 to 5.0, but did not study higher pH values than 5.0 [18]. In combination with the results of Zimmeck et al., the present findings suggest that 3-DG formation peaks at a slightly acidic pH and drops again at a neutral pH. To prove this hypothesis, further experiments investigating the whole pH scale are necessary. The contents of glyoxal and MGO, however, increased with the pH, but only the effect on MGO was statistically significant (Fig. 5b). The pH value is a critical factor for MGO formation. Until now, the formation of MGO is mainly described by a retro-aldol cleavage of glucose via the 2,3-enole intermediate [32]. This reaction is enhanced at neutral or basic pH values and thus, more MGO is formed at higher pH values.

In accordance with the literature, the concentrations of total GDPs as well as individual GDPs were significantly lower in a control with pH 3.1 compared to the contents at pH 5.5 and 7.5 (Fig. 5a/b) [1]. Since the GDP concentrations were generally lower, DTPA had only slight effects on the levels of total and individual GDPs (Supplementary Fig. S4).

Our experiments revealed that the pH value has considerable influence on the heat-induced formation of α-dicarbonyls. Raising the pH value from 5.5 to the physiological pH value of 7.5 may significantly reduce the GDP load in PDFs, with the exception of glyoxal and MGO. At this point, it is difficult to speculate about the clinical significance of this result. Further studies are required that also include monocarbonyl GDPs such as acetaldehyde or hydroxymethylfurfural. Furthermore, possible clinical disadvantages of GDPs increasing at pH 7.5, mainly MGO, must be weighed against potential benefits by lower levels of GDPs decreasing at pH 7.5, mainly 3-DG, 3,4-DGE, glucosone and 3-DGal. A previous study, for example, indicated that the adverse effects of GDPs in PDFs are strongly dependent on their structure and concentrations. Thus, 3,4-DGE was the main determinant of PDF-mediated cytotoxicity and impairment of enzyme activity [8]. A similar structure- and concentration-dependent assessment of different GDPs must be performed for further clinically relevant parameters. The lowest levels of GDPs are formed at pH 3.1, which is clinically utilized by the application of double-chamber bag PDFs.

### Influence of metal ions on the GDP formation in PDFs at pH 7.5

**Fig. 6:**
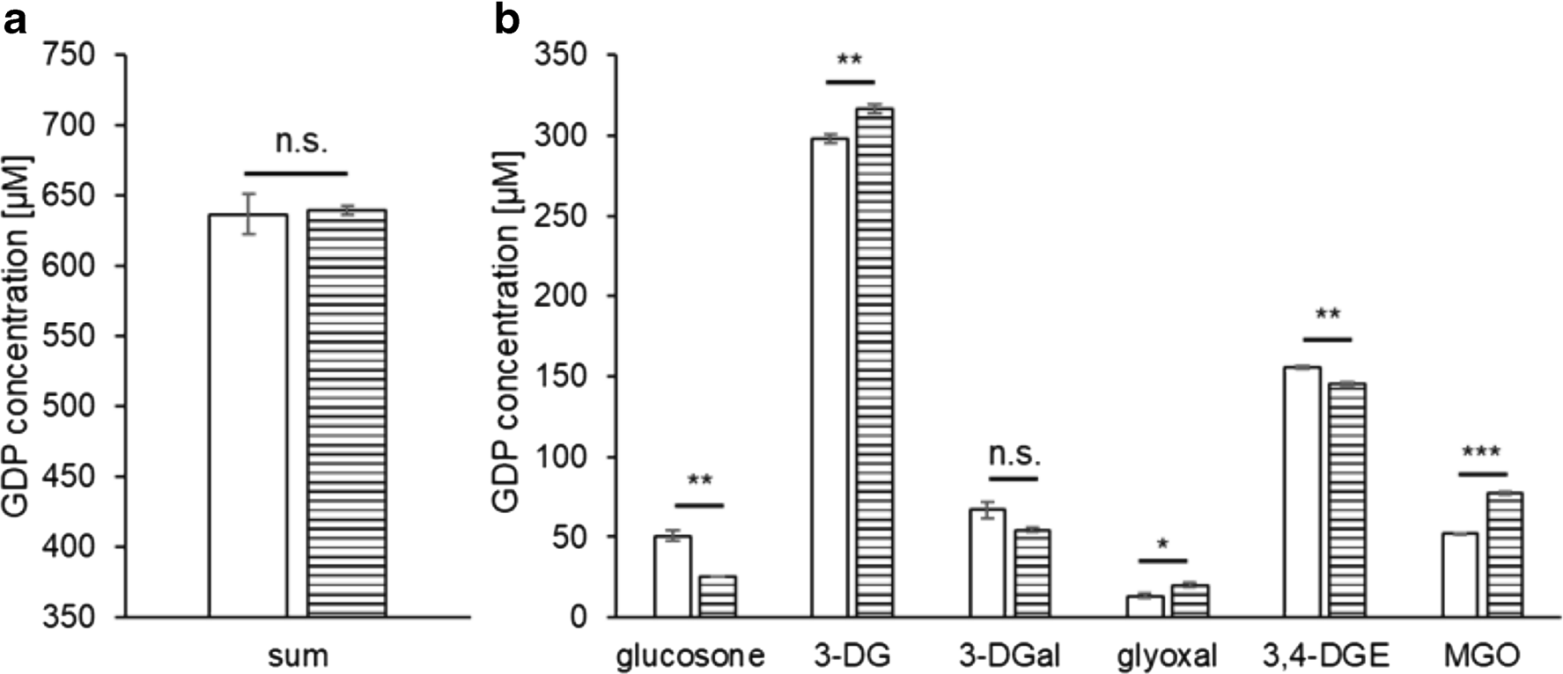
Concentrations of (A) total and (B) individual GDPs in heat-sterilized PDFs containing 4.25% glucose and conventional electrolytes at pH 7.5 without (white bars) and with DTPA (lined bars). Mean values ± standard deviations of triplicates are displayed. A two-tailed, paired t-test was calculated to show differences between the samples (n.s. not significant, * p < 0.05, ** < p 0.01, *** p < 0.001)

The influence of trace impurities of metal ions on the GDP formation was also investigated in PDFs at pH 7.5. At this pH, the addition of the chelator DTPA did not change the total GDP load (Fig. 6a), but had remarkable influence on the formation of individual GDPs. The formation of glucosone, 3-DGal and 3,4-DGE was reduced when the metal impurities were complexed by DTPA (Fig. 6b), although the effect was not significant for 3-DGal. Concentrations of 3-DG, glyoxal, and MGO, however, increased in the presence of DTPA.

**Fig. 7:**
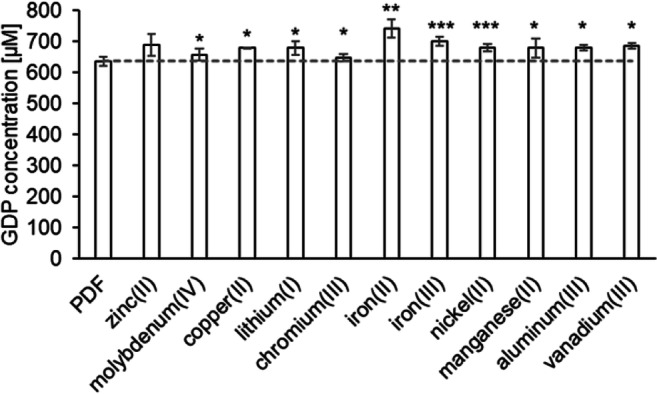
Concentrations of total GDPs in heat-sterilized unspiked PDF (pH 7.5, 4.25% glucose; control) and the same fluid spiked with different metal cations. The dashed line shows the GDP level of the control fluid. Mean values ± standard deviations of triplicates are displayed. A two-tailed, paired t-test was calculated to show differences between the control (PDF) and the samples (* p < 0.05, ** p < 0.01, *** p < 0.001)

**Fig. 8:**
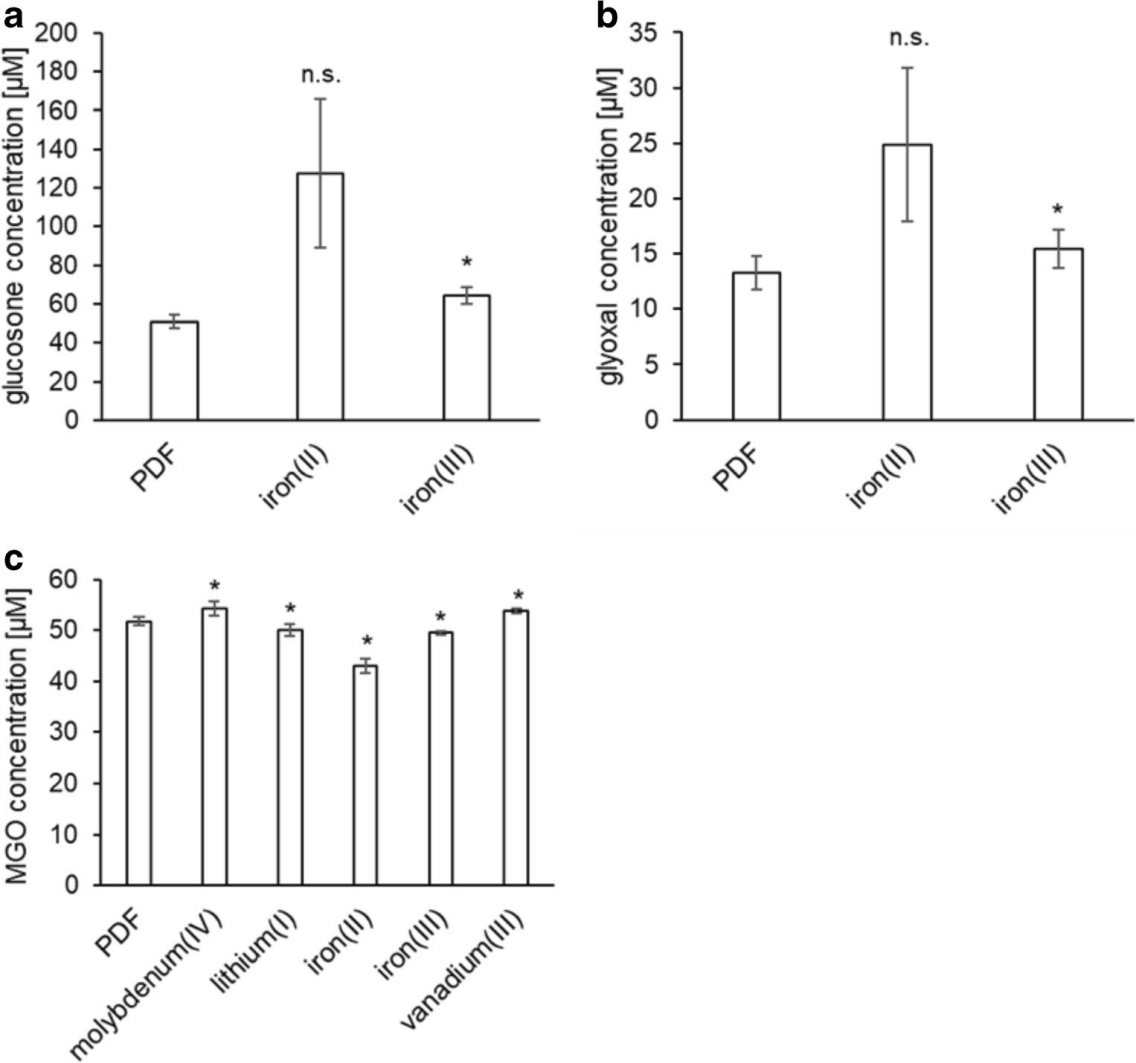
Concentrations of individual GDPs in an unspiked PDF (pH 7.5, 4.25% glucose; control) and the same fluid spiked with different metal cations. Mean values ± standard deviations of triplicates are displayed. A two-tailed, paired t-test was calculated to show differences between the control (PD) and the samples (* p < 0.05, ** p < 0.01). Results with non-significant differences are displayed in the electronic supplementary material (Figs. S5/6)

To investigate how specific metal ions affect the GDP formation at pH 7.5, the spiking experiments described above were repeated with PDFs at pH 7.5. The total GDP content was increased by all eleven inorganic cations (Fig. 7). Iron(II) approximately doubled the concentration of glucosone (128 μM vs. 62 μM) and glyoxal (25 μM vs. 11 μM), but the effect was not significant due to the high variation of results (Fig. 8a/b). Iron(III) led to a moderate but significant increase of glucosone and glyoxal. All other metal cations did not influence the contents of glucosone or glyoxal (Supplementary Fig. S5a/b). The formation of MGO increased significantly in the samples containing additional molybdenum(IV) or vanadium(III) and decreased when lithium(I), iron(II), and iron(III) were present (Fig. 8c). All other metals did not affect the formation of MGO (Supplementary Fig. S5c). A slight but significant increase in the formation of 3-DGal could be observed in the presence of zinc(II), copper(II), lithium(I), iron(III), nickel(II), or vanadium(III) (Supplementary Fig. S6a).The 3-DG and 3,4-DGE content also increased when the solutions were spiked with copper(II), lithium(I), chromium(III), iron(III), nickel(II), aluminum(III), or vanadium(III) (Supplementary Fig. S6b/c). Additionally, zinc(II) and manganese(II) led to an elevated 3-DG content (Supplementary Fig. S6b).

The experiments revealed that the impact of the individual metal ions on the GDP formation is pH dependent. This finding was expected for catalytic effects of redox-active cations, because redox potentials are pH dependent [42]. Interestingly, some metal ions also influenced the formation of non-oxidative GDPs such as 3-DG or 3-DGal. Further studies will be necessary to investigate the detailed reaction mechanisms underlying this observation.

The main effects on GDP formation were caused by iron(II), manganese(II), and chromium(III). It should not be left unmentioned that these metal cations were also applied at the highest concentration levels, so that a concentration-dependent effect might also contribute to the present findings. All inorganic cations and the respective concentration levels were selected on the basis of a metal screening to emulate the situation in commercial PDFs. Therefore, it can be assumed that these three metal cations have major impact on the GDP profile of PDFs, either because of their higher prevalence in the fluids or due to specific catalytic mechanisms.

## Conclusion

In summary, it was shown that the addition of the chelating agent DTPA reduced the α-dicarbonyl formation during the heat sterilization of PDFs significantly. This observation indicates that metal ions considerably enhance the degradation of glucose. Whereas the formation of α-dicarbonyls originating from dehydration processes like 3-DG, 3-DGal, or 3,4-DGE was not promoted, the contents of glucosone, glyoxal, and MGO increased in the presence of pro-oxidative cations. The metal traces in PDFs result from raw material contaminants or from production equipment made of stainless steel. Therefore, the purity of the raw materials can be an important quality parameter for the production of PDFs to reduce the formation of GDPs. Additionally, even trace contaminations with inorganic cations should be prevented throughout the production process. Alternatively, traces of metal ions could be inactivated by chelating agents. Finally, the current results indicate that heat sterilization at a physiological pH value of 7.5 may improve the biocompatibility of PDFs and may slightly reduce the GDP formation.

## Supplementary Information

ESM 1 (PDF 513 kb)

## Abbreviations

GDP: glucose degradation product
PDF: peritoneal dialysis fluid
AGE: advanced glycation product
3-DG: 3-deoxyglucosone
3-DGal: 3-deoxygalactosone
3,4-DGE: 3,4-dideoxyglucosone-3-ene
MGO: methylglyoxal
ICP–MS: inductively coupled-plasma mass spectrometry
DTPA: 2-[bis[2-[bis(carboxymethyl)amino]ethyl]amino]acetic acid
