# Supplementary material for: Metal cations promote α-dicarbonyl formation in glucose-containing peritoneal dialysis fluids
Source: Glycoconj J. 2020 Dec 7;38(3):319–29. doi: 10.1007/s10719-020-09964-6 (PMC8116238; doi:10.1007/s10719-020-09964-6)
Supplement: Supplementary file 1 — (PDF 513 kb) [file 10719_2020_9964_MOESM1_ESM.pdf]

Supplementary Material

Metal cations promote α-dicarbonyl formation in glucose-containing peritoneal dialysis fluids

Sabrina Gensberger-Reigl[1], Andrea Auditore[1], Jochen Huppert[2], Monika Pischetsrieder [1*]

[1] Food Chemistry, Department of Chemistry and Pharmacy, Friedrich-Alexander-Universität Erlangen-Nürnberg (FAU), Nikolaus-Fiebiger-Straße10, 91058 Erlangen, Germany.

[2] Fresenius Medical Care Deutschland GmbH, Frankfurter Straße 6-8, 66606 St. Wendel, Germany

A

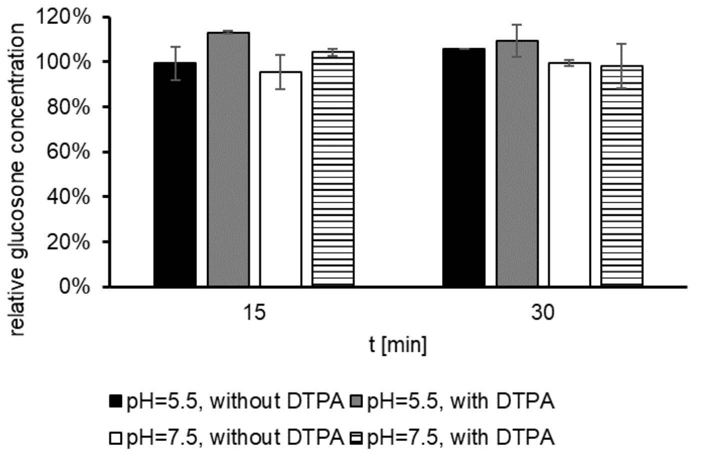

120%
100%
80%
60%
40%
20%
0%
relative glucosone concentration
15
30
t [min]
pH=5.5, without DTPA
pH=5.5, with DTPA
pH=7.5, without DTPA
pH=7.5, with DTPA

B

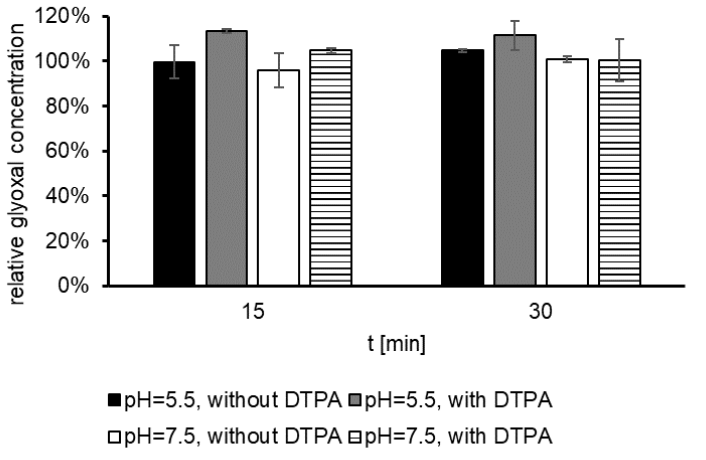

120%
100%
80%
60%
40%
20%
0%
relative glyoxal concentration
15
30
t [min]
pH=5.5, without DTPA
pH=5.5, with DTPA
pH=7.5, without DTPA
pH=7.5, with DTPA

C

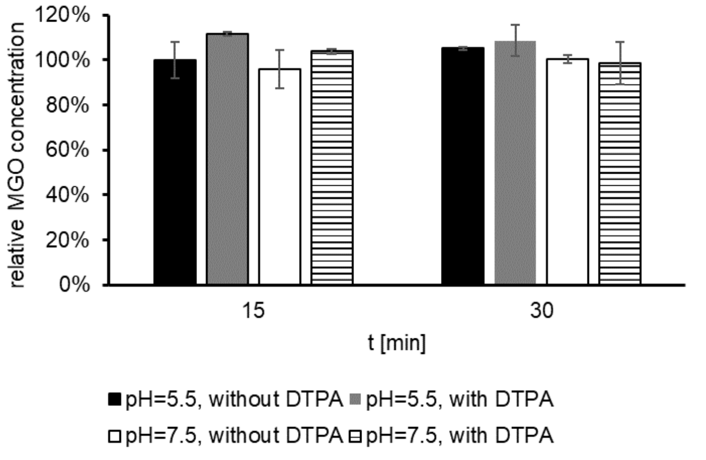

120%
100%
80%
60%
40%
20%
0%
relative MGO concentration
15
30
t [min]
pH=5.5, without DTPA
pH=5.5, with DTPA
pH=7.5, without DTPA
pH=7.5, with DTPA

D

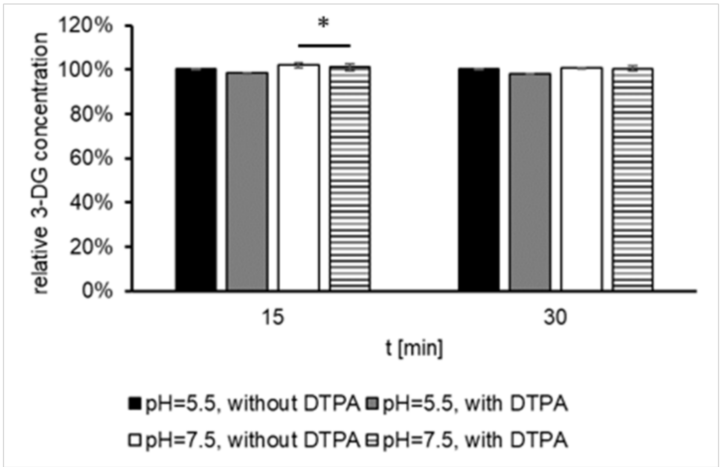

E

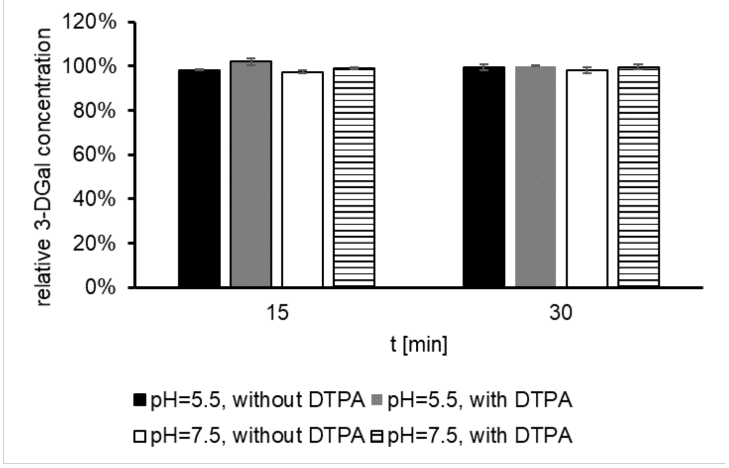

F

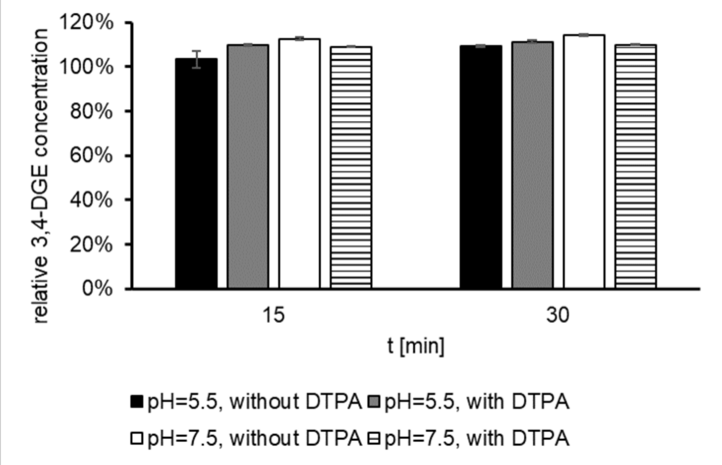

Fig. S1 Stability of GDPs at pH 5.5 and 7.5 with and without DTPA (A, glucosone; B, glyoxal, C, methylglyoxal, D, 3-deoxyglucosone, E, 3-deoxygalactosone, F, 3,4-DGE). Solutions of the GDPs were derivatized directly or 15 and 30 min after sterilization. The concentrations at 0 min were used as reference (100%). Mean values ± standard deviations of triplicates are displayed. A two-tailed, paired t-test was calculated to show differences between the samples with and without DTPA (*$p < 0.05$). Statistically significant differences were not observed unless marked.

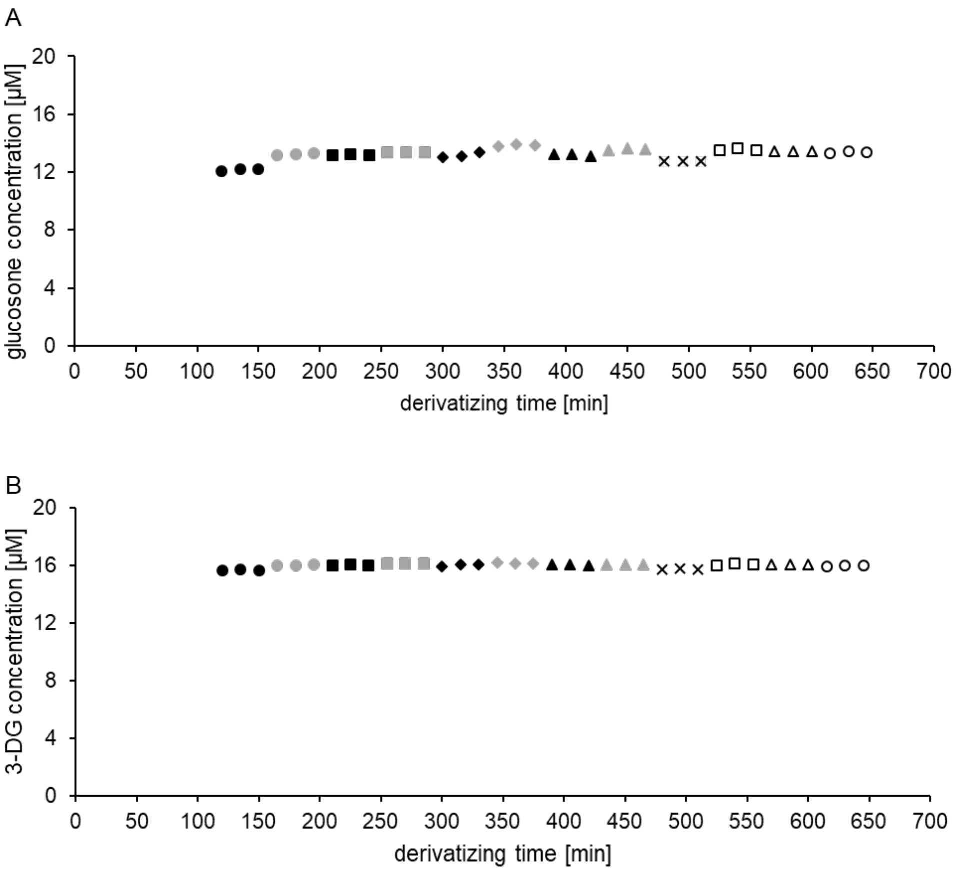

Fig. S2 Effect of the derivatizing time on the concentration of (A) glucosone and (B) 3-DG in different unheated PDFs at pH 5.5 containing conventional electrolytes and additional metal cations. The samples contained either zinc(II) (●), molybdenum(II) (●), copper(II) (■), lithium(I) (■), chromium(III) (♦), iron(II) (♦), iron(III) (▲), manganese(II) (X), nickel(II) (▲), vanadium(II) (□), aluminum(III) (Δ), or no additional inorganic cations (○).

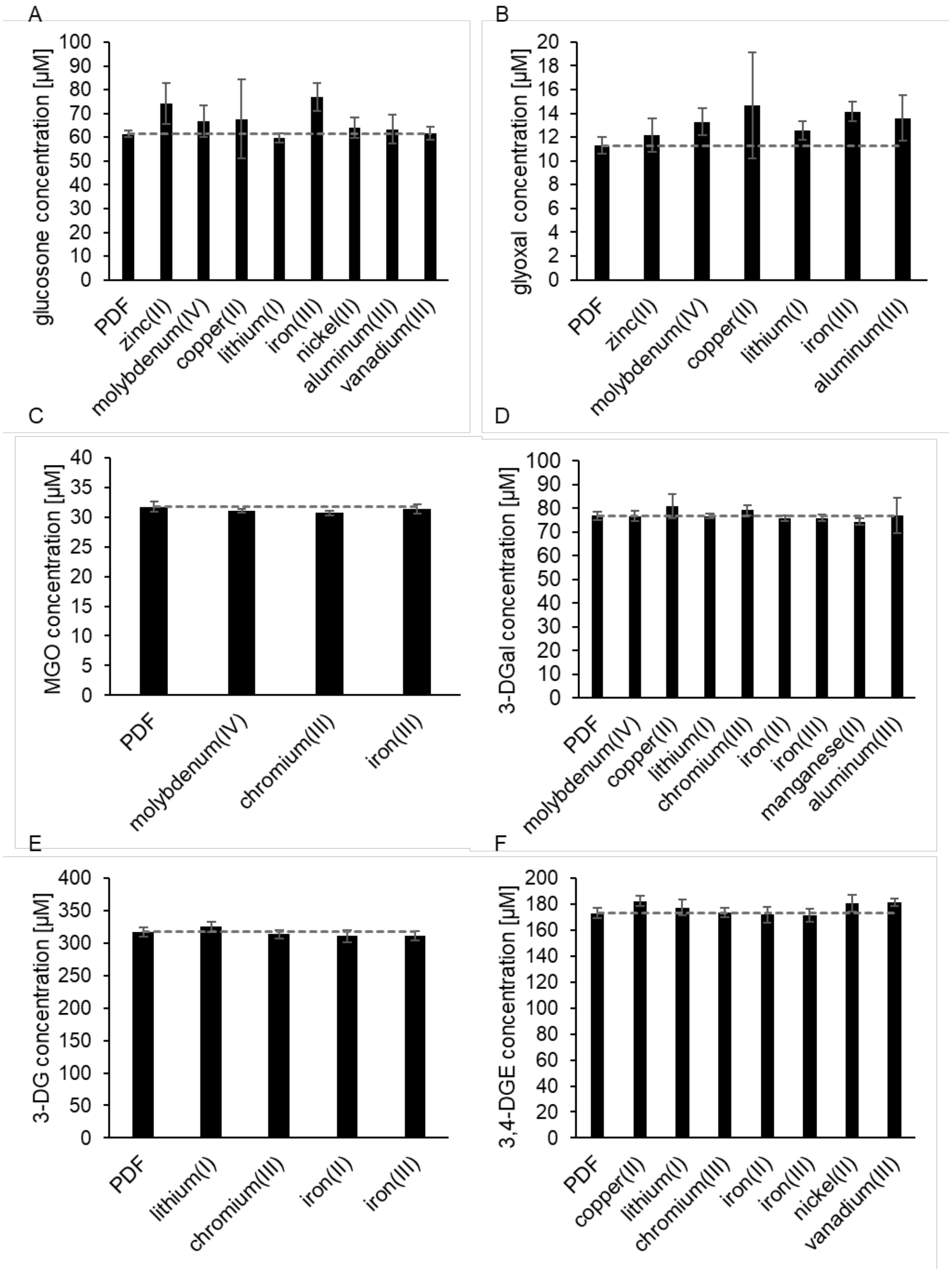

Fig. S3 Concentration of six GDPs in PDF (pH 5.5, 4.25% glucose; control) and the same fluid spiked with different metal cations. The diagrams show non-significant differences. Mean values ± standard deviations of triplicates are displayed. A two-tailed, paired t-test was calculated to show differences between the control and the spiked PDF.

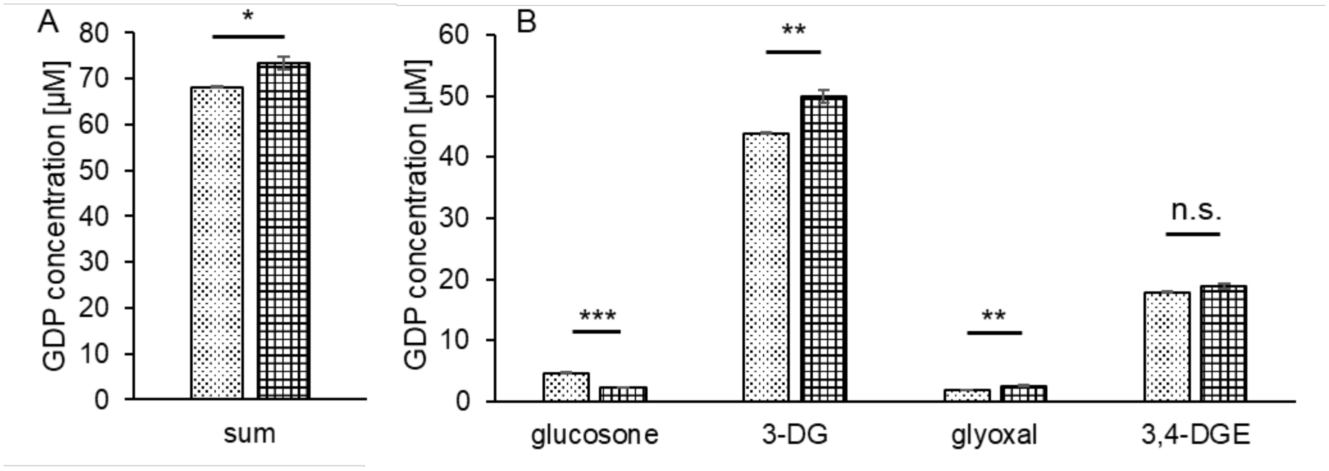

Fig. S4 Concentrations of (A) total and (B) individual GDPs in heat-sterilized PDFs containing 4.25% glucose and conventional electrolytes at pH 3.1 with DTPA (grid bars) and without DTPA (dotted bars). Mean values ± standard deviations of triplicates are displayed. A two-tailed, paired t-test was calculated to show differences between the samples with and without DTPA (n.s. not significant, * $p < 0.05$, *** $p < 0.001$). At pH 3.1, 3-DGal coeluted with 5-hydroxmethylfurfural so that it could not be quantified in these samples. MGO was below the limit of quantification.

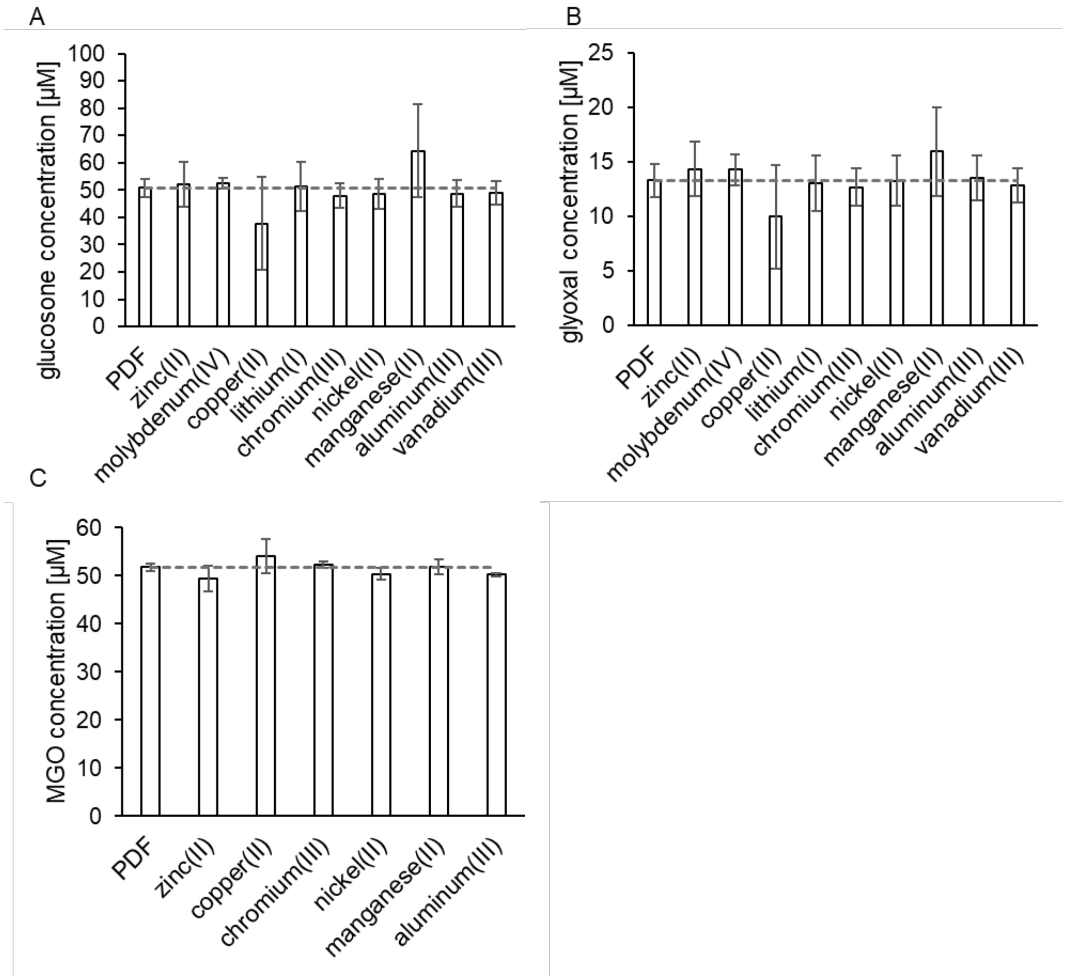

Fig. S5 Concentration of (A) glucosone, (B) glyoxal, and (C) MGO in PDF (pH 7.5, 4.25% glucose; control) and the same fluid spiked with different metal cations. The diagrams show non-significant differences. Mean values ± standard deviations of triplicates are displayed. The dashed line shows the GDP level of the control fluid. A two-tailed, paired t-test was calculated to show differences between the control and the spiked PDFs.

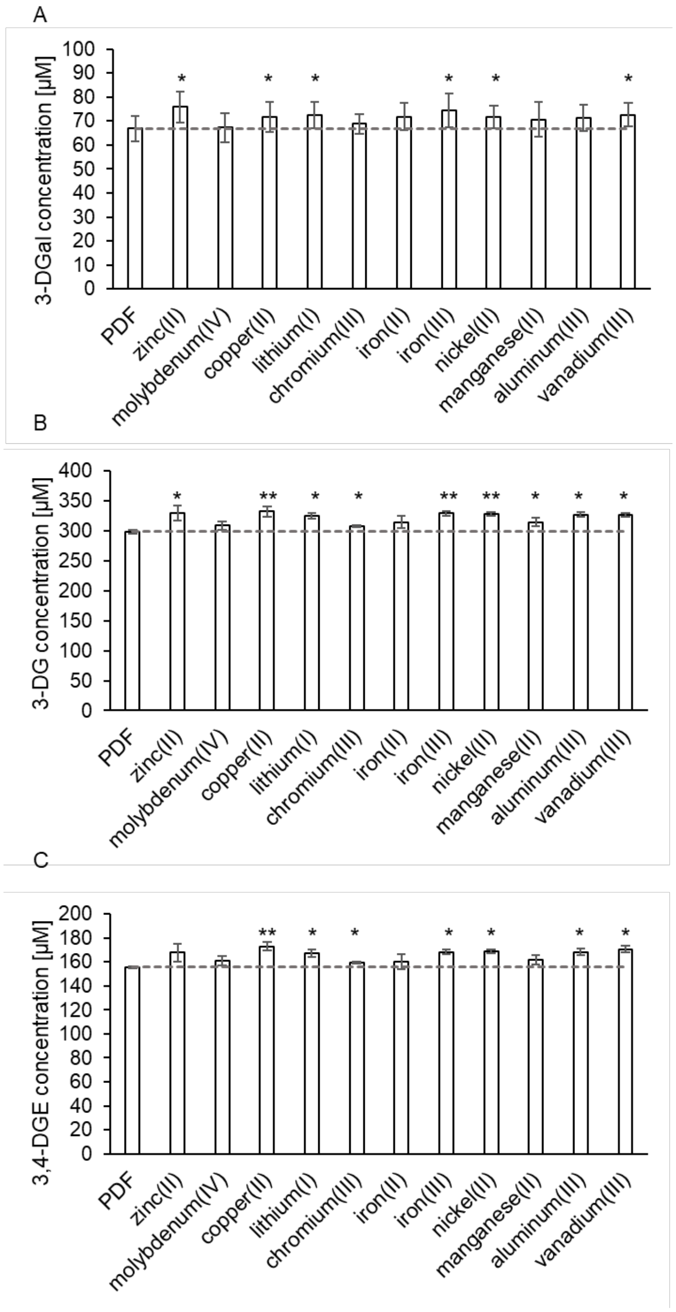

Fig. S6 Concentration of (A) 3-DGal, (B) 3-DG, and (C) 3,4-DGE in PDF (pH 7.5, 4.25% glucose; control) and the same fluid spiked with different metal cations. Mean values ± standard deviations of triplicates are displayed. A two-tailed, paired t-test was calculated to show differences between the samples and the control at pH 7.5 (* $p < 0.05$, ** $p < 0.01$). The dashed line shows the GDP level of the control fluid.
